# A potassium‐chloride co‐transporter with altered genome architecture functions as a suppressor in glioma

**DOI:** 10.1111/jcmm.18352

**Published:** 2024-04-29

**Authors:** Hongwei Liu, Zhouyang Pan, Xuelei Lin, Long Chen, Qi Yang, Wei Zhang, Luohuan Dai, Yihao Zhang, Wang Li, Yinhua Chen, Kang Peng, Siyi Wanggou, Feiyue Zeng, Xuejun Li

**Affiliations:** ^1^ Department of Neurosurgery, Xiangya Hospital Central South University Changsha China; ^2^ Hunan International Scientific and Technological Cooperation Base of Brain Tumor Research, Xiangya Hospital Central South University Changsha China; ^3^ Department of Radiology, Xiangya Hospital Central South University Changsha China

**Keywords:** GABA, glioma, immunotherapy, microenvironment, topologically associating domain

## Abstract

Gliomas, the most lethal tumours in brain, have a poor prognosis despite accepting standard treatment. Limited benefits from current therapies can be attributed to genetic, epigenetic and microenvironmental cues that affect cell programming and drive tumour heterogeneity. Through the analysis of Hi‐C data, we identified a potassium‐chloride co‐transporter SLC12A5 associated with disrupted topologically associating domain which was downregulated in tumour tissues. Multiple independent glioma cohorts were included to analyse the characterization of SLC12A5 and found it was significantly associated with pathological features, prognostic value, genomic alterations, transcriptional landscape and drug response. We constructed two SLC12A5 overexpression cell lines to verify the function of SLC12A5 that suppressed tumour cell proliferation and migration in vitro. In addition, SLC12A5 was also positively associated with GABA_A_ receptor activity and negatively associated with pro‐tumour immune signatures and immunotherapy response. Collectively, our study provides a comprehensive characterization of SLC12A5 in glioma and supports SLC12A5 as a potential suppressor of disease progression.

## 1 INTRODUCTION

Gliomas, especially glioblastoma (GBM), are the most common and incurable brain cancers, with median survival of 15–18 months in patients.^1^ Current treatments including maximal surgical resection followed by radiotherapy, chemotherapy, tumour treatment field and immunotherapy have not yet shown durable clinical benefits for glioma patients.^2^ Limited benefits from these therapies can be attributed to genetic, epigenetic and microenvironmental cues that affect cell programming and drive tumour heterogeneity.^3^ Identifying potential targets involved in aforementioned factors may be helpful for gaining further insight into the mechanisms of glioma progression.

The three‐dimensional genome organization of cancer cells is reprogrammed during tumorigenesis with accumulated alterations at the level of compartments, topologically associating domains (TADs), or chromatin loop.^4^ Distant regulatory elements can interact with target promoters via three‐dimensional looping, in which the majority of loops occurring between two regions are located within the same TAD.^5^ Therefore, TAD may act as barriers to facilitate or prevent loop interactions leading to differential gene expression and the role of genes located in these altered regions remains to be interrogated in detail.

Solute Carrier Family 12 Member 5 (SLC12A5) encodes a potassium‐chloride co‐transporter 2 (KCC2) that is responsible for the maintenance of low intracellular chloride concentrations through transporting chloride ions outwards from cells.^6^ The oncogenic function for SLC12A5 has been reported in colorectal cancer, bladder urothelial carcinoma and prostate cancer that upregulation of SLC12A5 can promote tumour progression and increase cell invasion and metastasis ability in vivo and in vitro.^7^, ^8^, ^9^ However, the exact function of SLC12A5 in gliomas and the mechanisms by which it affects tumour progression remain unclear.

In this study, we reported the identification of genome‐structurally altered SLC12A5 based on Hi‐C (high throughput chromosome conformation capture) data and it could function as a suppressor in glioma correlated with patient prognosis, GABA_A_ receptor activity, immune‐enriched microenvironment and immunotherapy response.

## 2 MATERIALS AND METHODS

### 2.1 Cell culture

Human normal astrocytes (HA1800) and glioma‐derived cell lines (A172, U87, U251, HS683 and LN229) were obtained from the Cancer Research Institute of Central South University. HA1800 were cultured using Astrocyte Medium (ScienCell) supplemented with 1% astrocyte growth supplement and 10% fetal bovine serum (FBS). Glioma‐derived cell lines were cultured in DMEM (Bio‐Channel) supplemented with 10% FBS. All cells were maintained at 37°C with 5% CO_2_.

### 2.2 Lentivirus transfection and generating stable cell lines

SLC12A5 overexpression lentivirus (OE‐KCC2) was designed and synthesized by GENECHEM. The sequence of SLC12A5 obtained from NCBI was cloned into GV492 vector and empty vector (OE‐NC) was used as negative control. Cells were transfected with lentivirus for 24 h and selected for positive cells with 2 μg/mL puromycin. Besides, the transfection efficiency of SLC12A5 overexpression was observed and analysed using fluorescence microscope.

### 2.3 Real‐time quantitative PCR

Total RNAs for clinical glioma samples and each cell line were extracted according to the manufacturer's instructions of FlashPure Total RNA Mini Kit (GeneBetter). And then, 1 μg of total RNA were reverse transcribed into cDNA using TransScript® Uni All‐in‐One cDNA Synthesis Kit (TransGen). Real‐time quantitative PCR (qPCR) was performed on QuantStudio™ Real‐Time PCR System (Applied Biosystems) with 2× BRYT Green qPCR Master Mix (Promega). Designed primers were as follows: SLC12A5 (forward sequence: GCAGGAGCCATGTACATCCT; reverse sequence: CCATGCAGGTGAGCACACA); GAPDH (forward sequence: CTCCTGCACCACCAACTGCT; reverse sequence: GGGCCATCCACAGTCTTCTG). GAPDH were used as internal control for gene expression and ΔΔCT method was utilized to quantify relative mRNA expression.

### 2.4 Immunofluorescence staining

After seeding cells in coverslips with appropriate number, cells were fixed with 4% PFA for 15 min and permeabilized using 0.1% TritonX‐100 for 15 min at room temperature. Then, blocking buffer (5% bovine serum albumin) was applied for 1 h at room temperature. Cells were incubated with primary rabbit anti‐KCC2 antibody (Abcam #ab259969, 1:200) or primary mouse anti‐Ki67 antibody (Cell Signalling Technology #9449, 1:1000) overnight at 4°C. All antibodies were diluted in 1% blocking buffer. Secondary antibody conjugated to Alexa Fluor 555 (Invitrogen A32794; A32773) was used at 1:1000 for 1 h in dark at room temperature. Finally, DAPI was added to stain the nucleus and incubated in dark for 5 min. Images were taken using Leica DM6B microscope and quantified using ImageJ software.

### 2.5 Wound healing assay

After cells reached 90% confluence in 6‐well plate, the cells were wounded by scratching with a 100 μL sterile pipette tip which was perpendicular to the bottom of the plate. Impaired cells were subsequently eliminated through washing by phosphate buffered saline (PBS). Then, DEME without FBS were added to plate for excluding the influence of cell proliferation. Finally, position and size of the scratches were recorded by microscope under low magnification at 0 h, 24 h and 48 h. The ratio of 24 h or 48 h wound area to 0 h wound area in each group was calculated by ImageJ software.

### 2.6 Public data sets and clinical samples retrieval

Processed Hi‐C data of A172 and HA1800 were from our previous study, in which detailed analysis methods could be found and TAD boundary was identified using intra‐chromosomal Hi‐C matrices at 50 kb resolution.^10^ TCGA glioma ATAC‐seq and somatic mutation data, TCGA pan‐cancer RNA‐seq including lower grade glioma (LGG) and GBM and GTEX RNA‐seq with their clinical data, were downloaded from the University of California, Santa Cruz (UCSC) Xena through the online data website (https://xenabrowser.net/). Two batches of CGGA RNA‐seq including CGGA_325 (CGGA1) and CGGA_693 (CGGA2) with their clinical data were obtained from the CGGA website (http://www.cgga.org.cn/). GLASS RNA‐seq data set and its survival information of glioma patients were downloaded via Synapse (https://www.synapse.org/#!Synapse:syn17038081/files/). In addition, other glioma cohorts or cell line transcripts (GSE16011, GSE43289 and GSE15824) and a single‐cell RNA‐seq data (GSE131928) were also obtained from GEO database. One GBM immunotherapy‐related cohort (PRJNA482620) including 14 samples with pre‐therapy and post‐therapy state was obtained from the SRA database.^11^ Finally, normal brain tissue (*n* = 3) and glioma samples (*n* = 3 in each grade) were collected from the neurosurgery department of Xiangya Hospital of Central South University to detect the expression of SLC12A5.

### 2.7 Sequencing data processing

Raw sequencing data of PRJNA482620 were mapped to the hg38 reference genome through HISAT2 (version 2.2.1) and all the transcript data were log normalized. For single cell data, we applied R package Seurat (version 4.0.3) to normalized the expression following features select and dimensionality reduction. Same marker profiles of the cell type reported in the original article were compared with our current study to define cell type. Cells with the count of a particular feature exceeding 1 were defined as ‘feature‐positive cells’. Normalized ATAC‐seq of enhancer peak signals (log2((count+5)PM)‐qn) for TCGA glioma had been processed in the source database. Somatic mutation data in this study were processed by R package maftools (version 2.8.05).

### 2.8 Gene set enrichment analysis and subtype classification

Tumour microenvironment signatures including anti‐tumour, pro‐tumour, angiogenesis and fibrosis, and malignant cell properties were generated by Bagaev, et al.^12^ Immune evasion signatures based on 19 genes related to markers of T‐cell dysfunction were download from previous study.^13^ Synaptic gene set used contained PTPRS, ARHGEF2, GRIK2, DNM3, LRRTM2, GRIK5, NLGN4X, NRCAM, MAP2, INA and TMPRSS9 as previous study done.^14^ Gene set of TGF‐beta pathway was obtained from MSigDB (http://software.broadinstitute.org/gsea/msigdb). We used single sample enrichment analysis (ssGSEA) algorithm in R package GSVA (version 1.40.1) and AddModuleScore function in Seurat to estimate the enrichment of each signature in bulk transcript data sets and single‐cell data sets respectively. All calculated scores were scaled to range 0–1. Additionally, Gene Ontology (GO) and Kyoto Encyclopedia of Genes and Genomes (KEGG) enrichment analysis were performed using R package clusterProfiler (version 4.7.1). Subtypes of glioma sample including classical (CL), mesenchymal (MES) and proneural (PN) were identified by R package ssgsea.GBM.classification.^15^ While four main cellular states in single cell level containing neural‐progenitor‐like (NPC‐like), oligodendrocyte‐progenitor‐like (OPC‐like), astrocyte‐like (AC‐like) and mesenchymal‐like (MES‐like) states were identified by previous defined calculated method.^16^

### 2.9 Differential gene identification and survival analysis

According to median expression level of SLC12A5, patients were divided into SLC12A5 high‐expression group and low‐expression group. Then, differential expression genes (DEGs) between these two groups were identified using R package limma (version 3.48.0) with false discovery rate (FDR) <0.01 and|log2(fold change)|>1.0 as cut off value. To determine the prognostic value of SLC12A5, we used R package survival (version 3.3–1) and survminer (version 0.4.9) to calculate hazard ratios (HRs) with 95% confidence intervals (CI) and log‐rank *p* values in survival analysis and used ezcox (version 1.0.2) to perform multivariate Cox proportional hazard regression analysis.

### 2.10 Drug response predicting

We download the drug sensitivity measured by half maximal inhibitory concentration (IC50) and gene expression profiles for cancer cell lines from Genomics of Drug Sensitivity in Cancer (GDSC) (https://osf.io/c6tfx/). Then we used R package oncoPredict (version 0.2) to predict clinical drug response in TCGA glioma cohorts based on GDSC data set as a training set with default parameters.^17^ The Spearman's rank correlation between SLC12A5 expression and IC50 was calculated to assess imputed drug sensitivity and|correlation coefficient|>0.3 and *p* value<0.05 were considered as statistically significant.

### 2.11 Tumour purity estimation and immune infiltration analysis

In order to infer tumour purity from gene expression data, we used ESTIMATE algorithm to calculate purity score, which combined both a stromal and immune gene set together by ssGSEA.^18^ In addition, we also used CIBERSORT algorithm to deconvolute infiltrated immune cell types in tumour sample.^19^

### 2.12 Statistical analysis

R software (version 4.1.3) and Adobe Photoshop CS6 software were used to conduct data analysis and graph generation. Protein intensity in immunofluorescence staining were quantified by ImageJ software. Continuous variables fitting a normal distribution between binary groups were compared using a two‐tailed Student's t test, otherwise Wilcoxon test. Continuous variables between multiple groups were compared using Kruskal–Wallis test or one‐way ANOVA test. Group differences of categorical variables was investigated using chi‐square test. Correlations between variables were explored using Pearson or Spearman coefficients. *P* < 0.05 was considered statistically significant.

## 3 RESULTS

### 3.1 Hi‐C identifies a putative target gene SLC12A5 associated with disrupted topologically associating domain

**FIGURE 1:**
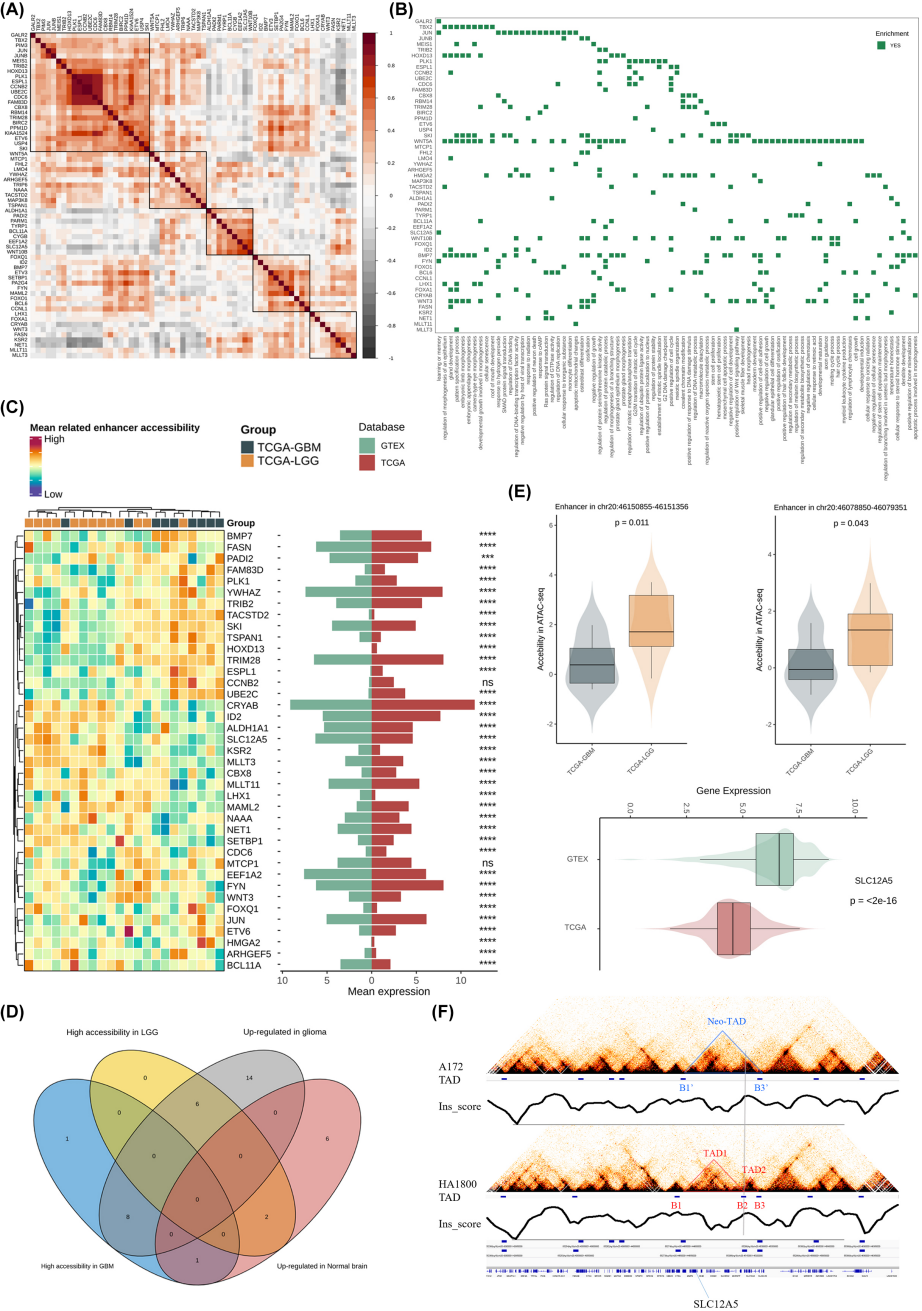
A putative target gene SLC12A5 is associated with disrupted topologically associating domain. (A) Pearson correlation among the expression of 64 potential genes derived from Hi‐C data in TCGA glioma. (B) Biological process enrichment of 64 potential genes in GO. (C) Left: Accessibility of gene‐related enhancer peak between TCGA‐GBM and TCGA‐LGG. Right: Mean expression of each gene between TCGA glioma tissue and GTEX normal brain tissue. *p < 0.05, **p < 0.01, ***p < 0.001 and ****p < 0.0001, ns: no statistics. (D) Venn diagram displaying intersections of genes with differential accessibility of enhancer peak and expression level between TCGA glioma tissue and GTEX normal brain tissue. (E) Top: Specific enhancer peak signals of SLC12A5 between TCGA‐GBM and TCGA‐LGG. Bottom: Expression level of SLC12A5 between TCGA glioma tissue and GTEX normal brain tissue. (F) Altered TAD structures where SLC12A5 is located between A172 and HA1800 cell line based on Hi‐C data.

In our previous study, we explored alterations in three‐dimensional chromatin organization between glioma‐derived cell line (A172) and normal astrocytes (HA1800) by Hi‐C.^10^ Due to the functions of TAD that protected genes from abnormal regulation of regulatory elements outside TAD, we identified total 64 potential genes located in disrupted TADs between A172 and HA1800 excluding known oncogenes and tumour suppressors, which had various transcriptional patterns in glioma cohorts (Figure 1A). The majority of these genes were associated with cell mitotic process, cell morphogenesis, DNA packaging, cell–cell signal transduction and other biological activities of cell growth (Figure 1B). To determine whether any of these genes showing different accessibility of their related enhancers, we investigated the enhancer peak signal in the ATAC‐seq of TCGA glioma. Interestingly, parts of genes, such as TSPAN1 and TRIM28, had higher accessibility of enhancer region in GBM compared to LGG and were significantly upregulated in tumour tissue than normal brain tissue (Figure 1C). While only two genes including SLC12A5 and KSR2 exhibited lower accessibility of enhancer region in GBM and decreased level of expression in glioma tissue (Figure 1D). SLC12A5, also named KCC2, were a potassium‐chloride co‐transporter and associated with brain network activity and nervous system development (Figure 1B). Two predicted‐enhancer peaks of SLC12A5 in chromatin 20 were more open in LGG than GBM and its expression were also obviously decreased in glioma tissue indicating its tumour suppressor role in glioma (Figure 1E). Specifically, the boundary B2 of HA1800 vanished in A172, causing the merging of TAD1 and TAD2 into neo‐TAD, consequently altering regulatory elements of SLC12A5 (Figure 1F).

### 3.2 Clinicopathological characteristics of SLC12A5 revealed its role as a potential target in glioma

**FIGURE 2:**
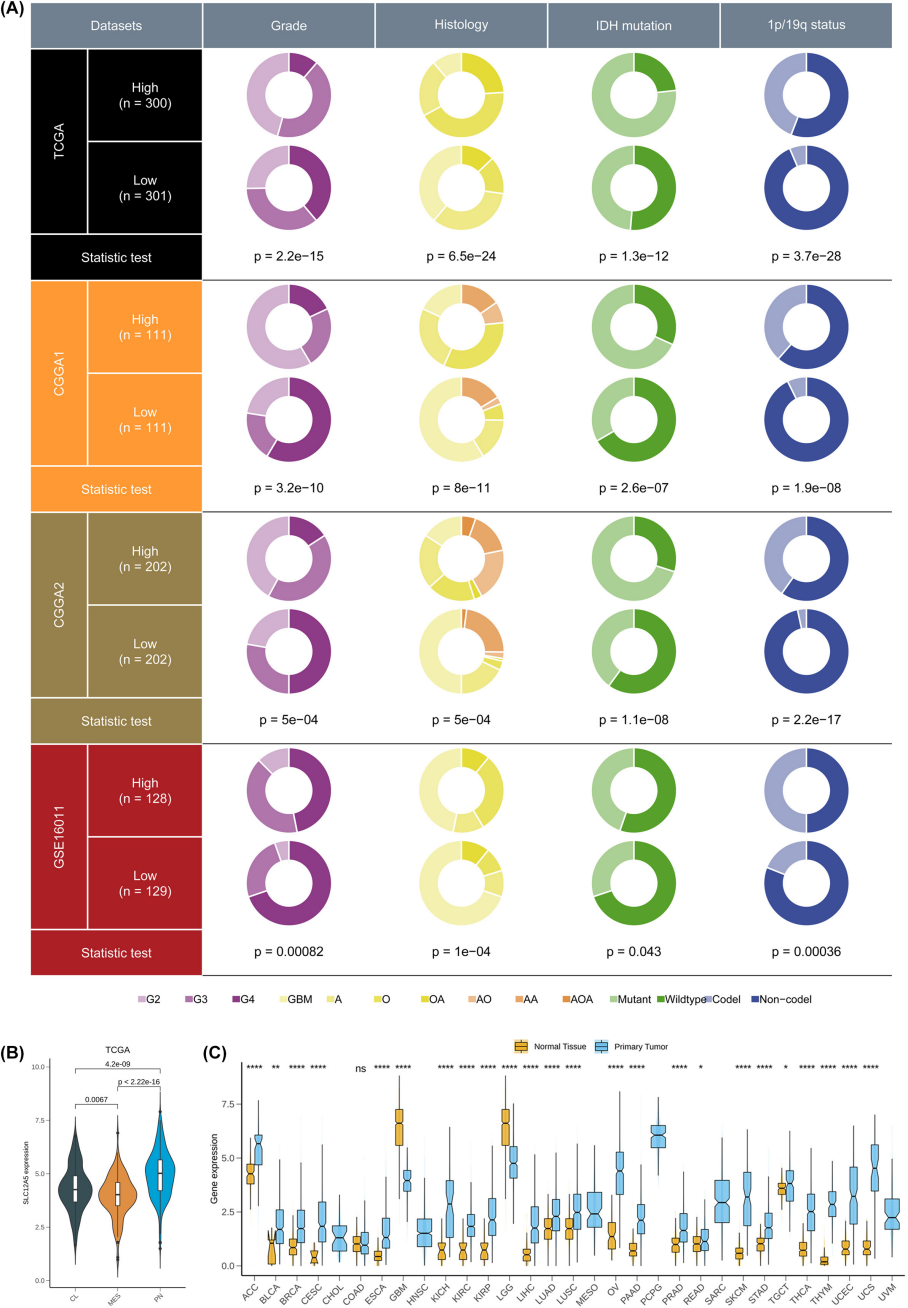
Clinicopathological characteristics of SLC12A5 in glioma. (A) The relationship between expression of SLC12A5 and other clinical features (Grade, Histology, IDH mutation and 1p/19q status) in TCGA, CGGA1, CGGA2 and GSE16011 data sets. Statistic test: chi‐square test. (B) Expression level of SLC12A5 among CL, PN and MES subtypes in TCGA glioma. (C) Expression level of SLC12A5 between tumour and normal tissue in pan‐cancer data sets. *p < 0.05, **p < 0.01 and ***p < 0.001, ****p < 0.0001, ns: no statistics.

We further investigated the relationship between expression of SLC12A5 and other clinical features in four independent cohorts. In TCGA glioma cohorts, low expression of SLC12A5 was significantly associated with increased tumour grade, IDH wild‐type and 1p/19q non‐codeletion status which were known molecular biomarkers (Figure 2A, Figure S1A). Histologically, SLC12A5 showed the lowest expression in GBM according to the WHO Classification system (Figure 2A, Figure S1A). Similarly, we validated these findings in three other cohorts and got consistent results (Figure 2A, Figure S1B–D). These results suggested that the expression of SLC12A5 decreased gradually with the increased malignant degree of glioma. As molecular subtypes were applied to differ survival ability in glioma patients where PN and CL had better prognosis than MES subtype,^15^ we also investigated their associations and found that MES subtype preferred low expression level of SLC12A5 in all four cohorts (Figure 2B, Figure S1E). Since the prognosis of elderly glioma is known to be poor, we explored the relationship between the expression of SLC12A5 and age of patients, which showed that the expression of SLC12A5 was weakly correlated with age of patients in four data sets indicating its independence (Figure S1F). To comprehensively analyse the function of SLC12A5 in various cancer types, we also explored its expression level between tumour and normal tissue in pan‐cancer data sets (Figure 2C). The expression of SLC12A5 in most of tumour tissues had significantly different degrees of changes compared to normal tissues suggesting that it might have a different biological mechanisms involved in progression of tumour among various cancer types.

### 3.3 Low expression of SLC12A5 predicts worse prognosis in glioma

**FIGURE 3:**
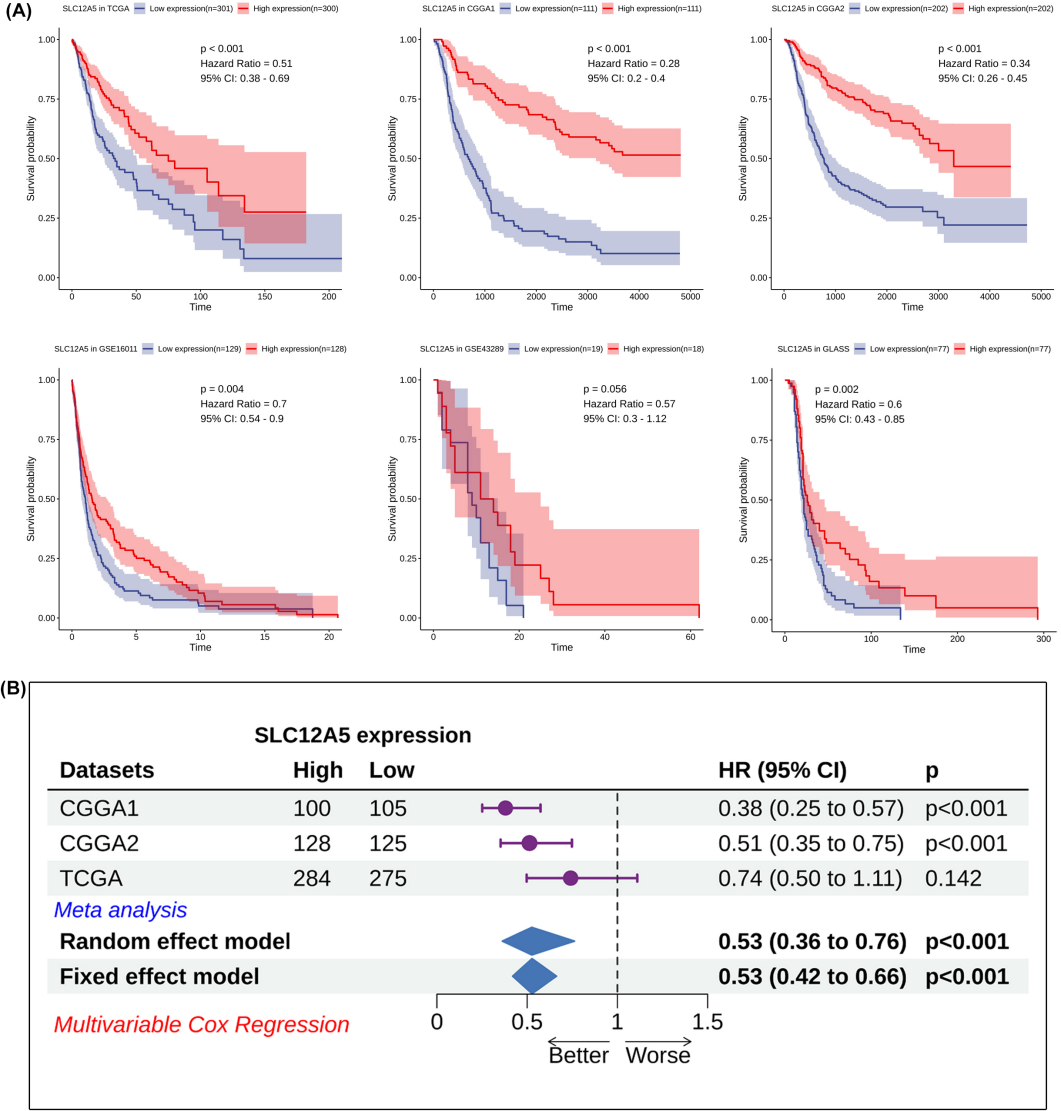
Prognostic value of SLC12A5 in glioma. (A) Correlation between SLC12A5 and overall survival of glioma patients in six cohorts. (B) Meta‐analysis of multivariate cox results from three data sets using random effect and fixed effect model.

Since SLC12A5 was overexpressed in low‐grade glioma and better prognostic subtypes, we set out to investigate the prognostic value of SLC12A5 across six independent cohorts. Intriguingly, patients with higher expression of SLC12A5 presented longer overall survival (OS) in all glioma cohorts with significant log‐rank test in five of six data sets (Figure 3A). The absence of significant statistic in only one cohort may be due to limitations in the number of patients. Meanwhile, we performed multivariate COX regression analysis to determine whether SLC12A5 was an independent prognostic factor by adjusting for other variables, including gender, age, WHO grade, IDH mutation status and MGMT methylation status (Figure S2A–C). Through meta‐analysis for combining cox results of three data sets, it demonstrated that SLC12A5 could be used as an independent prognostic factor for overall survival in glioma patients (HR = 0.53, *p* < 0.001) (Figure 3B). Additionally, pan‐cancer survival analysis showed that SLC12A5 could also functioned as a risk factor for OS in prostate adenocarcinoma (PRAD), liver hepatocellular carcinoma (LIHC) and kidney renal clear cell carcinoma (KIRC) (Figure S2D). Thus, we speculated that SLC12A5 had great application value in predicting the prognosis of glioma patients.

### 3.4 SLC12A5 contributes to distinct genomic alterations and transcriptomic landscapes

**FIGURE 4:**
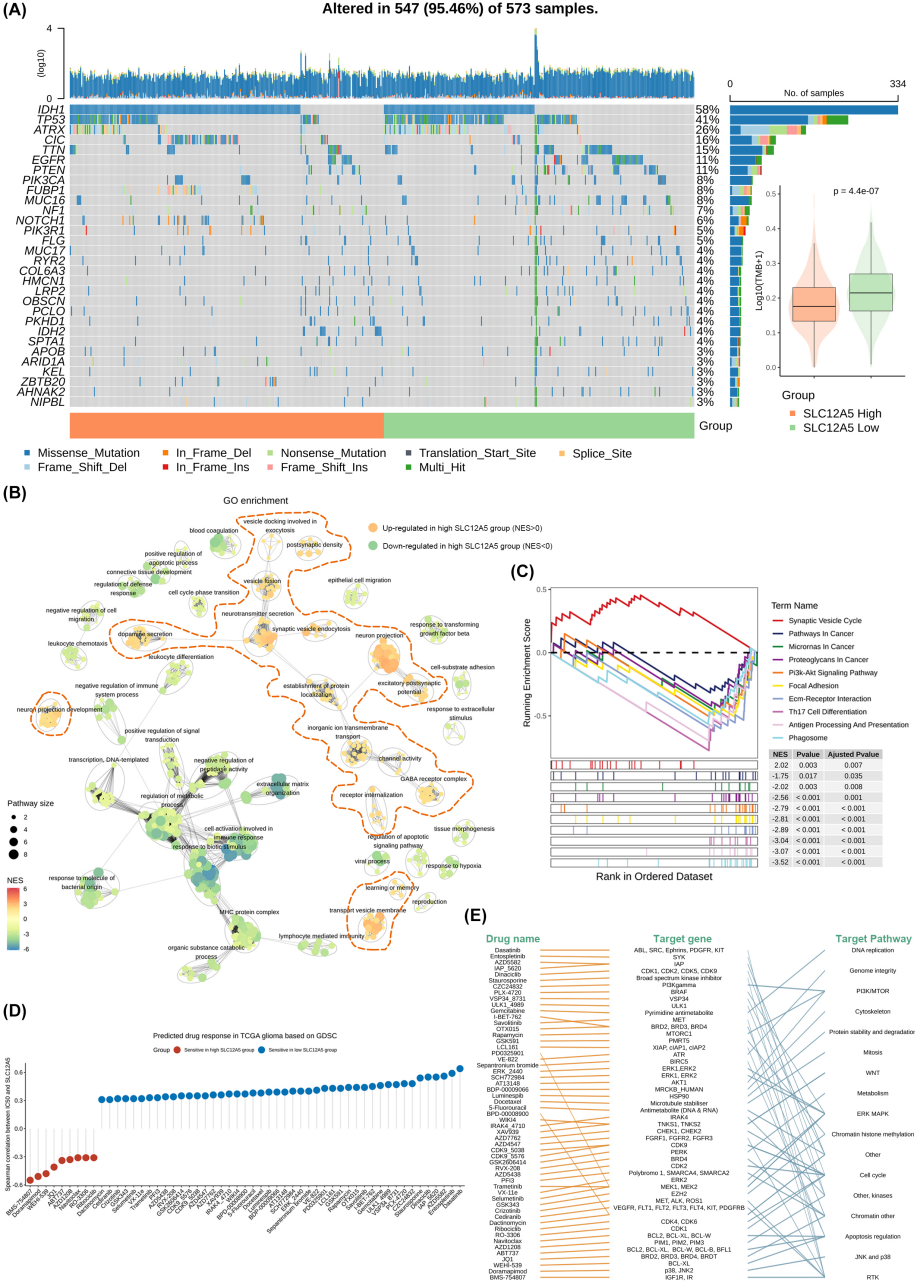
Characterization of genomic alterations, transcriptome features and drug response correlated with SLC12A5. (A) Landscape of somatic mutation between SLC12A5 high‐expression group and low‐expression group. (B) Network of enriched GO categories based on DEGs between these two groups (*Q* value <0.05). Normal Enrichment score (NES) >0 indicates upregulated processes in SLC12A5 high‐expression group. (C) KEGG enrichment results of DEGs between these two groups (*Q* value <0.05). (D) Spearman's correlation between SLC12A5 and predicted IC50 of drugs. Positive correlation means drug sensitive in SLC12A5 low‐expression group. (E) Targeted genes and pathways of drug listed in Figure 4D.

As somatic mutations could explain some molecular mechanisms involved in tumour progress, we classified patients in TCGA glioma cohort into two groups according to the median expression of SLC12A5 and compared the frequency of mutations between high‐expression group and low‐expression group. A higher frequency of mutations in low SLC12A5 expression group was detected indicating higher tumour mutation burdens which may be beneficial for immunotherapy because of more neo‐antigens production (Figure 4A). IDH1, as an essential biomarker in glioma patients, had missense mutation in most case of high SLC12A5 expression group, consistent with previous studies that glioma patients with IDH mutation had better outcomes. Besides, low SLC12A5 expression group had the most frequent mutations in TP53, ATRX, TTN, EGFR and PTEN. Of these most frequently altered genes, TP53 and PTEN are known tumour suppressors in glioma.^20^ Given the large genomic alterations that existed between two groups, we further explored the potential biological processes associated with SLC12A5 through the transcriptome (Figure S3A). Gene Ontology enrichment map showed that upregulated genes in high SLC12A5 group participated in ion channel activity, GABA receptor complex, excitatory postsynaptic potential and nervous system development, while processes about cell cycle, cell migration, apoptotic regulation, immune response and response to extracellular stimulate were downregulated (Figure 4B). Remarkably, pathways in cancer including PI3K‐Akt signalling, focal adhesion, ECM‐receptor interaction, antigen processing and phagosome were activated in tumour with low SLC12A5 expression (Figure 4C). These results strongly supported the correlation between low expression of SLC12A5 and tumour malignancy.

### 3.5 Integrative analysis of SLC12A5 on drug response

To further understand the effect of SLC12A5 on tumour malignancy and find potential drugs for therapy, we predicted drug sensitivity of total 198 drugs in TCGA glioma cohorts based on ridge regression model taking GDSC data sets as training set. The expression levels of SLC12A5 positively correlated with IC50 of most anticancer drugs suggested that these drugs may be sensitive in tumour with low SLC12A5 expression (Figure 4D). Notably, these drugs mainly targeted DNA replication, mitosis, cell cycle, apoptosis regulation, ERK MAPK pathway or PI3K/mTOR pathway, and activities of these pathways were simultaneously upregulated in low SLC12A5 expression group as analysed above (Figure 4E). For example, PI3K/Akt/mTOR pathway is a central regulator of cell proliferation, cell motility and chemoresistance in solid tumours, whose activation can lead to disease progression.^21^ AT13148, CZC24832 and rapamycin were all PI3K/AKT/mTOR inhibitors and rapamycin had been reported to suppress glioma cell growth by inhibiting PI3K‐Akt–mTOR signaling.^22^, ^23^, ^24^ Meanwhile, three targeted genes (AKT1, MTOR and PIK3CG) of these drugs showed significant upregulation in SLC12A5‐low tumour (Figure S3B).

### 3.6 Overexpression of SLC12A5 inhibit glioma cell proliferation and migration in vitro

**FIGURE 5:**
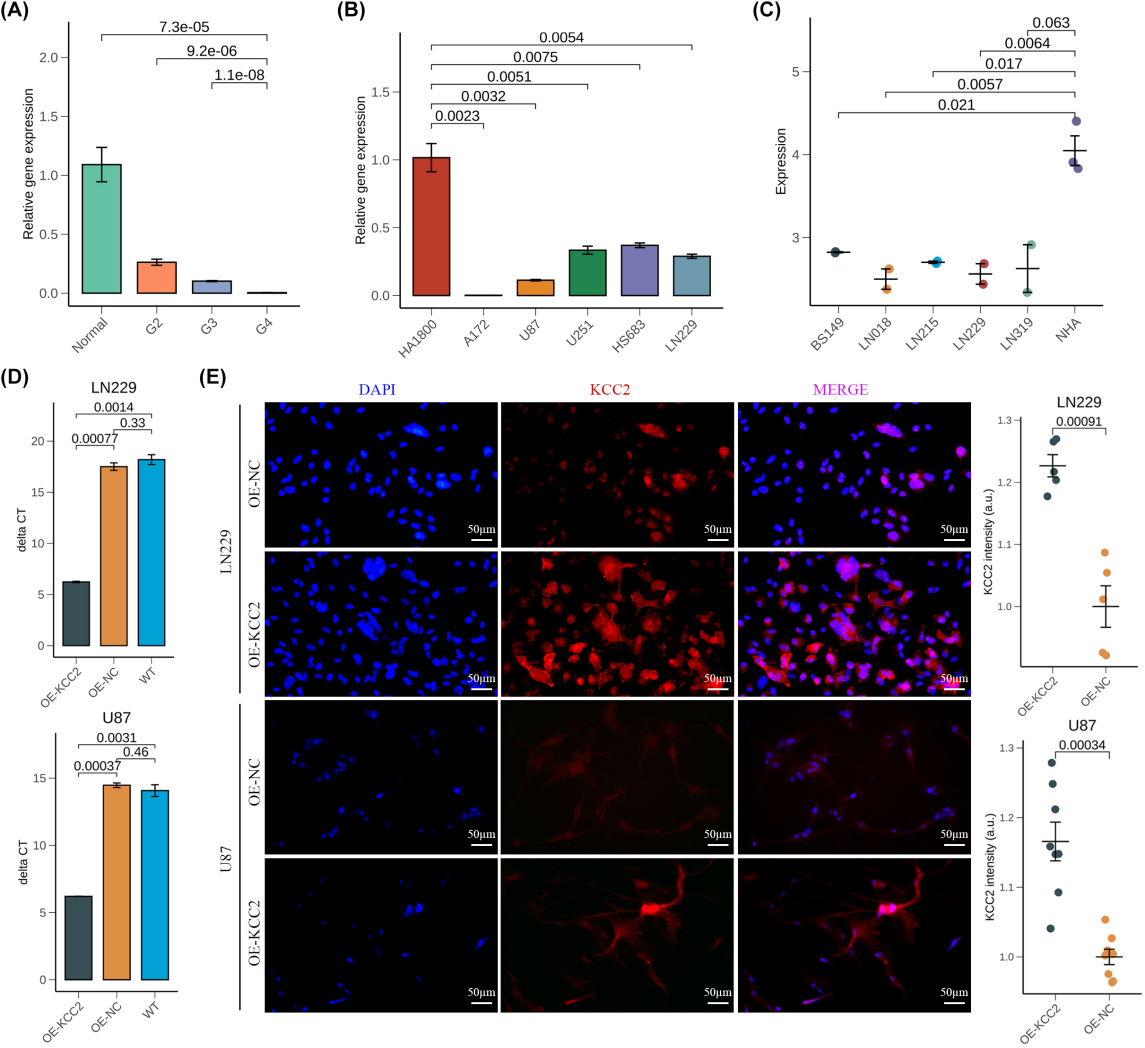
Construction of SLC12A5 overexpression cell lines. (A) Relative expression of SLC12A5 in clinical surgical samples by qPCR. Normal brain tissue: *n* = 3; Grade 2 glioma tissue: *n* = 3; Grade 3 glioma tissue: *n* = 3; Grade 4 glioma tissue: *n* = 3. (B) Relative expression of SLC12A5 in different cell lines by qPCR. (C) Expression level of SLC12A5 in GSE15824 data sets. Normal human astrocytes: NHA; Glioblastoma cell lines: LN018, LN215, LN229, LN319 and BS149. (D) Relative expression of SLC12A5 in LN229 and U87 cell lines under OE‐KCC2, OE‐NC and WT conditions by qPCR. OE‐KCC2: SLC12A5 overexpression; OE‐NC: negative control; WT: wild type. (E) Left: representative images of LN229 and U87 cell lines under OE‐KCC2 and OE‐NC conditions labelled by DAPI (Blue) and KCC2 (Red). Right: quantification of KCC2 intensity in different conditions.

In order to validate our findings above, we evaluated whether SLC12A5 was differentially expressed in clinical surgical samples and different glioma cell lines. By quantitative reverse transcriptase polymerase chain reaction, we detected that the expression of SLC12A5 gradually decreased with the increase of tumour grade and was higher in HA1800 than in other glioma cell lines (Figure 5A,B). Published transcription data from glioma cell lines also confirmed highest expression level of SLC12A5 in normal human astrocytes (Figure 5C). Based on these results, we conducted overexpression of SLC12A5 in LN229 and U87 cell line via lentivirus infection for further biological research. After lentivirus transduction, the transcription level of SLC12A5 in OE‐KCC2 condition of both cell lines was obviously higher than that in WT and OE‐NC conditions (Figure 5D). In addition, SLC12A5 protein levels were also significantly upregulated in both cell lines under OE‐KCC2 conditions (Figure 5E).

**FIGURE 6:**
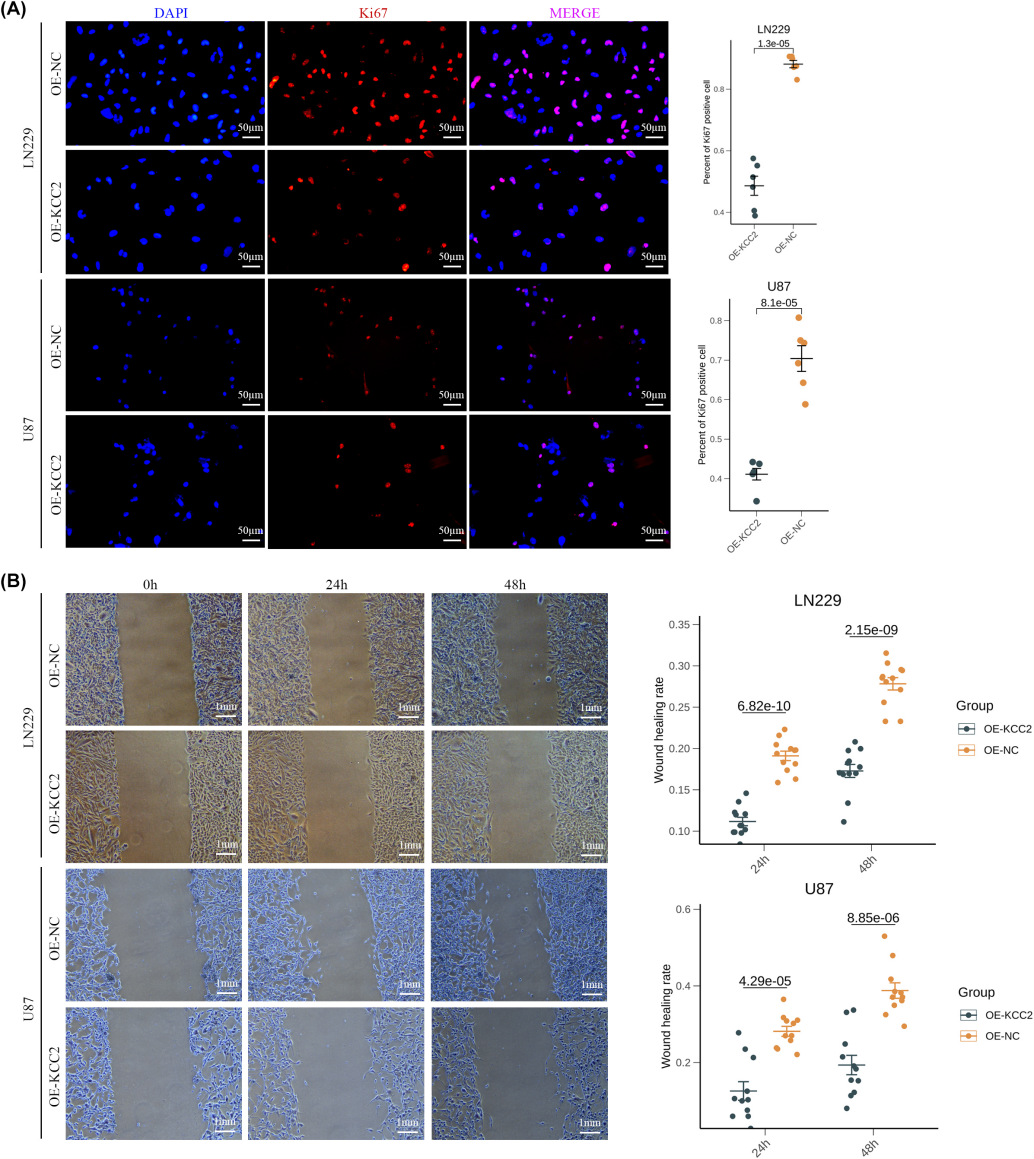
Overexpression of SLC12A5 suppresses glioma cell growth. (A) Left: Representative images of LN229 and U87 cell lines under OE‐KCC2 and OE‐NC conditions labelled by DAPI (Blue) and Ki67 (Red). Right: Quantification of Ki67‐positive cell number in different conditions. (B) Left: Wound healing area of LN229 and U87 cell lines under OE‐KCC2 and OE‐NC conditions. Right: Quantification of wound healing area in different conditions.

Due to the association between elevated level of SLC12A5 and downregulation of cell proliferation and migration, we sought to evaluate the effect of SLC12A5 on these biological behaviours of glioma cells. Ki67 staining showed that number of Ki67‐positive cells was all significantly decreased in LN229 and U87 cell line under OE‐KCC2 conditions (Figure 6A). Wound‐healing assays also demonstrated that overexpression of SLC12A5 could distinctly inhibit migration ability of LN229 and U87 cell line meanwhile (Figure 6B). Therefore, these data supported the tumour suppressor role of SLC12A5 in glioma consistent with our results from bioinformatics analysis.

### 3.7 Correlation of SLC12A5 with GABA_A_ receptor and synaptic activity

**FIGURE 7:**
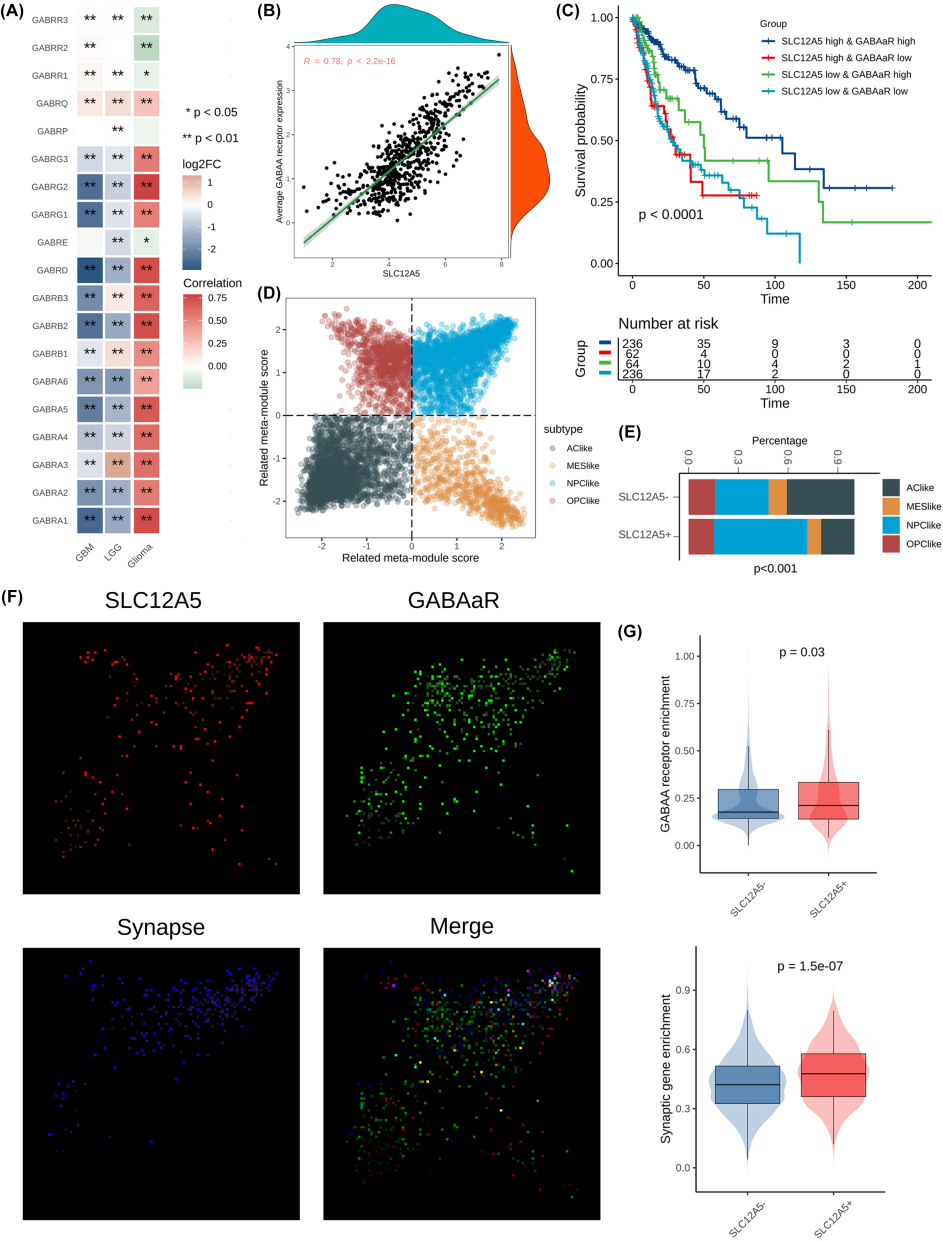
GABA_A_ receptor and synaptic activity may be involved in SLC12A5‐induced inhibition mechanisms. (A) Pearson coefficients between SCL12A5 and each GABA_A_ channel subunits in TCGA glioma cohorts. *p < 0.05 and **p < 0.01. (B) Correlation between SLC12A5 and average expression of GABA_A_ receptor based on 19 subunit‐related genes in TCGA glioma cohorts. (C) Survival analysis in TCGA glioma cohorts by combining the expression of GABA_A_ receptor and SLC12A5. (D) Four cellular states of tumour cell in single cell level based on related meta‐module score. (E) Different subtypes preference among SLC12A5‐positive and SLC12A5‐negative tumour cells. (F) The expression level of SLC12A5, GABA_A_ receptor and synaptic genes in single‐cell RNA‐seq. Red points mean cells with the count of SLC12A5 exceeding 1; blue points mean cells with the enrichment score of synaptic genes exceeding 90% quantiles of it in NPC‐like cells; green points mean cells with the enrichment score of GABA_A_ receptor exceeding 90% quantiles of it in NPC‐like cells; merge image is the superimposition of three colours. (G) Comparison of the enrichment score of GABA_A_ receptor and synaptic genes between SLC12A5‐positive and SLC12A5‐negative tumour cells.

After a comprehensive analysis of different biological processes related to SLC12A5, we found that GABA receptor and synaptic activity were upregulated when expression of SLC12A5 was elevated (Figure 4B). As gamma‐aminobutyric acid (GABA) was the main inhibitory neurotransmitter in the central nervous system and endogenous GABA_A_ receptor activity had been reported to suppresses glioma growth,^25^ we further investigated the association between GABA_A_ channel subunits and SLC12A5. Interestingly, the majority of GABA_A_ channel subunits were significantly positively correlated with SLC12A5 at the transcriptional level (Figure 7A). With the increased expression of SLC12A5, the average expression of GABA_A_ receptor was also increased (Figure 7B). By combining the expression of GABA_A_ receptor and SLC12A5 to divide glioma patients into four groups, we found that OS of patients could be obviously distinguished under this stratification in which patients with high expression of GABA_A_ receptor and SLC12A5 had the best outcome (Figure 7C).

Recent works had uncovered bidirectional interactions between neurons and tumours to drive tumour progression through electrochemical synapses and paracrine factors, and synapse‐related genes were mainly enriched in stem‐like cells and oligodendroglial precursors.^14^, ^26^ Thus, we attempted to determine the distribution of SLC12A5 across different cell types via single‐cell sequencing data and tumour cells were further classified into four subtypes based on modules defined by Neftel et al (Figure 7D, Figure S3C). Strikingly, SLC12A5 was mainly expressed in tumour cells and most specifically in NPC‐like tumour cells which characterized stem and progenitor cell signatures and highly expressed GABA_A_ receptor and synaptic genes (Figure 7E, Figure S3D–F). We thereafter evaluated the association between SLC12A5 and GABA_A_ receptor or synaptic gene at single cell level, and found that these features existed co‐expressed condition in individual cell, with SLC12A5‐positive tumour cells having higher expression of GABA_A_ receptor and synaptic genes than SLC12A5‐negative tumour cells (Figure 7F,G). SLC12A5 encodes a potassium‐chloride co‐transporter KCC2, which is a main Cl^−^ extruder and essential for maintaining GABA hyperpolarizing.^27^ Collectively, these data suggested that tumour‐induced SLC12A5 reduction may contribute to cell depolarization caused by neuronal excitability for tumour growth through reducing GABA's inhibitory signalling.

### 3.8 Low expression of SLC12A5 shape an immune‐enriched microenvironment and predict response to immunotherapy

**FIGURE 8:**
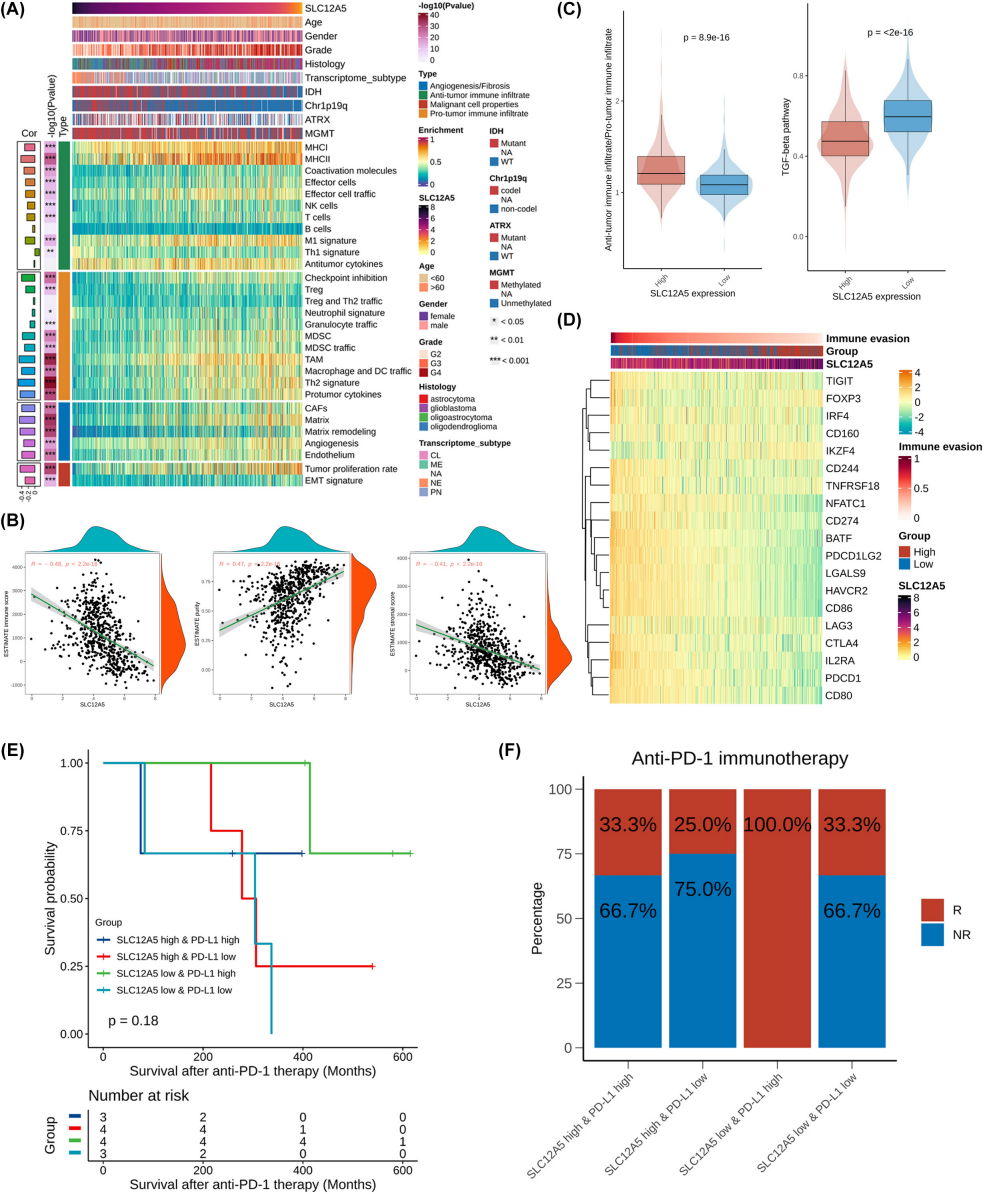
SLC12A5 contributes to tumour immune microenvironment and immunotherapy response. (A) eat map shows the correlation between SLC12A5 and tumour microenvironment features in TCGA glioma cohorts. CL, classical; ME, mesenchymal; NA, not applicable; NE, neural; PN, proneural; WT, wild type. (B) Correlation between SLC12A5 and immune score, stromal score and tumour purity calculated by ESTIMATE in TCGA glioma cohorts. (C) Comparison of anti/pro‐tumour immune infiltrate ratio and TGF‐beta pathway activity between SLC12A5 high‐expression group and low‐expression group in TCGA glioma cohorts. Anti‐tumour immune infiltrate: Average enrichment score of anti‐tumour immune signatures; Pro‐tumour immune infiltrate: Average enrichment score of anti‐tumour immune signatures. (D) Heat map shows the correlation between SLC12A5 and immune evasion signatures in TCGA glioma cohorts. (E) Survival analysis in a GBM cohorts accepted PD‐1 inhibitors treatment by combining the expression of PD‐L1 and SLC12A5. (F) Graphs show response efficacy of immunotherapy in four groups of GBM patients. NR, non‐responder; R, responder.

The significant correlations between SLC12A5 and immune system process suggested that SLC12A5 may be involved in altered tumour microenvironments (Figure 4B). We utilized 29 curated gene sets related to anti‐tumour microenvironment, pro‐tumour microenvironment, angiogenesis and fibrosis, and malignant cell properties, to assess the glioma microenvironment landscape by single‐sample gene set enrichment analysis (Figure 8A). Of note, tumour with low expression of SLC12A5 presented an immune‐enriched microenvironment with abundance of stromal and proliferative signatures (Figure 8A, Figure S4A). We obtained consistent results calculated by ESTIMATE algorithm in which SLC12A5 was significantly positively correlated with tumour purity while negatively correlated with immune and stromal score (Figure 8B). Decreased ratio of anti‐tumour immune infiltrate/pro‐tumour immune infiltrate in tumour with low expression of SLC12A5 indicated a more immunosuppressive microenvironment (Figure 8C). To validate this observation, we performed deconvolution to assess immune cell infiltrations using TCGA and CGGA data sets in which activated NK cells were significantly enriched in high SLC12A5 expression group while M2 macrophages were significantly enriched in low SLC12A5 expression group (Figure S4B). Fibroblasts are strong immune suppressors and tumour microenvironment remodellers by secreting TGF‐beta.^28^ Indeed, the activity of TGF‐beta pathway was also significantly upregulated in tumours with low expression of SLC12A5 (Figure 8C).

Since patients with immune‐favourable microenvironment and high tumour mutation burden benefited the most from immunotherapy especially immune checkpoint blockade therapy,^29^, ^30^ we further assessed the effect of SLC12A5 on immunotherapy response. The majority of T‐cell dysfunction markers were highly expressed in tumours with low expression of SLC12A5, which also had a higher immune evasion score demonstrating the potentials of SLC12A5 on predicting response to immunotherapy (Figure 8D, Figure S4C). Currently, the clinical efficiency of PD‐1/PD‐L1 inhibitors are widely estimated in various cancer types^31^ and we collected a GBM cohort of total 14 participants who accepted PD‐1 inhibitors treatment to investigate the association between SLC12A5 and immunotherapy response. Patients with high PD‐L1 expression had better prognosis, while patients with low SLC12A5 expression were more likely to respond to immunotherapy and had better outcome despite not meeting statistical requirements, possibly due to limited cohort size (Figure S4D–F). Intriguingly, dividing patients into four groups by combining PD‐L1 and SLC12A5 expression, we found that patients with high PD‐L1 expression and low SLC12A5 expression had the best prognosis and basically all responded to immunotherapy (Figure 8E,F). Together, these data showed that SLC12A5 was associated with immune infiltrations and had predictive value for immunotherapeutic response in glioma.

## 4 DISCUSSION

In this study, we discovered a potassium‐chloride co‐transporter SLC12A5 with altered genome architecture between HA1800 and A172, which is downregulated in high‐grade glioma and also as a protective factor contributed to outcome of patients. Moreover, overexpression of SLC12A5 in glioma‐derived cell lines can suppress tumour cell proliferation and migration. Due to the differences in TAD region of SLC12A5, we need CRISPR‐mediated deletion of TAD boundary in the future to determine that this chromatin structure controls the expression of SLC12A5 further influencing the malignance of tumour cell. Unfortunately, specific regulatory elements located in this TAD are undefined in this study because of lacking in other epigenetic data. In addition, three‐dimensional chromatin structure landscapes of other glioma cell or tissue still need to be illustrated to confirm our findings.

GABA is the major inhibitory neurotransmitter in the central nervous system and the most common type of GABA receptors is the GABA_A_ channel, consisting of eight different subunit types encoded by 19 genes.^32^ Distinct GABA_A_ channel subunits present various expression patterns in glioma and are associated with tumour histology and clinical outcome.^33^ Specifically, GABA_A_ receptor agonist muscimol can attenuate the progression of glioma by activating Cl^−^ currents.^25^ Another agonist, moxidectin, enhances the expression of GABA_A_ receptor and inhibits the growth of paediatric medulloblastoma.^34^ While potentiating Cl^−^ extrusion by enhancing, KCC2 has been shown to reduce chemoconvulsant‐induced epileptiform activity through controlling the polarity of GABAergic synaptic signalling under conditions of hyperexcitation.^35^ Moreover, phosphorylation of KCC2 at Thr^906^ and Thr^1007^ can inhibit KCC2 activity leading to developmental disruption with impaired GABA‐mediated signal.^36^ These data show that KCC2 is crucial for the function of GABA during neurogenesis. More recently, it has been reported that remote neuronal activity or proximally synapse formed by glioma–neuron interactions can drive glioma progression.^14^, ^26^, ^37^ Based on the role of chloride transporters that regulate inhibitory synaptic plasticity mediated by the neurotransmitter GABA,^38^ we hypothesize that SLC12A5 maintains the function of GABA receptor through regulating Cl^−^ gradient across the membrane in turn blocking neuronal excitability induced by glioma–neuron interactions. This may be the mechanism why overexpression of SLC12A5 in tumour cell suppress its proliferation and migration and low expression level of gene in high‐grade glioma, which need relatively extensive works to be validated in the future.

Tumour microenvironment contributes to therapeutic resistance in glioma which prefer an immune‐desert microenvironment with T‐cell dysfunction and M2 phenotypic transformation.^39^ Immune checkpoint inhibitors, a class of molecules that modulate activity of immune cell to block inhibitory signals and promote anti‐tumour effects, have been applied across several tumour types.^31^ One commonly targeted molecular is PD‐1, which binds with PD‐L1 causing T cell exhaustion and apoptosis and how to improve the efficacy of anti‐PD‐1 or anti‐PD‐L1 therapy have been widely explored.^40^ In our study, grouping strategies by combining the expression of PD‐L1 and SLC12A5, can obviously distinguish the prognosis of patients. Notably, patients with low expression of SLC12A5 and high expression of PD‐L1 are all successful to respond to anti‐PD‐1 treatment. However, the number of GBM patients accepted immunotherapy is insufficient to meet statistical requirements and we need more data to confirm it. Furthermore, underlying mechanisms by which SLC12A5 regulates the immunomicroenvironment of glioma remain to be explored.

## 5 CONCLUSIONS

In summary, we identified SLC12A5, a candidate gene in glioma, from Hi‐C data which was decreased in tumour tissue and negatively correlated with tumour grade and malignant biological processes validated in vitro. It was particularly noteworthy that SLC12A5 was positively associated with GABA_A_ receptor activity and low expression of SLC12A5 shaped an immunosuppressive microenvironment in glioma indicating its predictive value for glioma immunotherapy.

## AUTHOR CONTRIBUTIONS

**Hongwei Liu:** Conceptualization (equal); data curation (equal); formal analysis (equal); investigation (equal); project administration (equal); supervision (equal); validation (equal); visualization (equal); writing – original draft (equal); writing – review and editing (equal). **Zhouyang Pan:** Data curation (equal); formal analysis (equal); validation (equal); visualization (equal). **Xuelei Lin:** Data curation (equal); formal analysis (equal); validation (equal); visualization (equal). **Long Chen:** Data curation (equal); formal analysis (equal); validation (equal); visualization (equal). **Qi Yang:** Data curation (equal); formal analysis (equal); validation (equal); visualization (equal). **Wei Zhang:** Data curation (equal); formal analysis (equal); validation (equal); visualization (equal). **Luohuan Dai:** Data curation (equal); formal analysis (equal); validation (equal); visualization (equal). **Yihao Zhang:** Data curation (equal); formal analysis (equal); validation (equal); visualization (equal). **Wang Li:** Data curation (equal); formal analysis (equal); validation (equal); visualization (equal). **Yinhua Chen:** Data curation (equal); formal analysis (equal); validation (equal); visualization (equal). **Kang Peng:** Data curation (equal); formal analysis (equal); validation (equal); visualization (equal). **Siyi Wanggou:** Resources (equal); supervision (equal); writing – review and editing (equal). **Feiyue Zeng:** Conceptualization (equal); resources (equal); supervision (equal); writing – review and editing (equal). **Xuejun Li:** Conceptualization (equal); funding acquisition (equal); resources (equal); supervision (equal); writing – review and editing (equal).

## FUNDING INFORMATION

This work is supported by the National Natural Science Foundation of China (Grant No. 82270825).

## CONFLICT OF INTEREST STATEMENT

All authors declared that the research was conducted in the absence of any commercial or financial relationships that could be construed as a potential conflict of interest.

## Supporting information


Figure S1.

Figure S2.

Figure S3.

Figure S4.

## Data Availability

Data used to support the findings of this study were all public and available from the corresponding author on reasonable request. This study did not generate custom code and new sequencing data for the analyses. Standard workflows and open‐source software used were described in Materials and Methods.
