# Supplementary figures and images for: A potassium‐chloride co‐transporter with altered genome architecture functions as a suppressor in glioma
Source: J Cell Mol Med. 2024 Apr 29;28(9):e18352. doi: 10.1111/jcmm.18352 (PMC11058328; doi:10.1111/jcmm.18352)

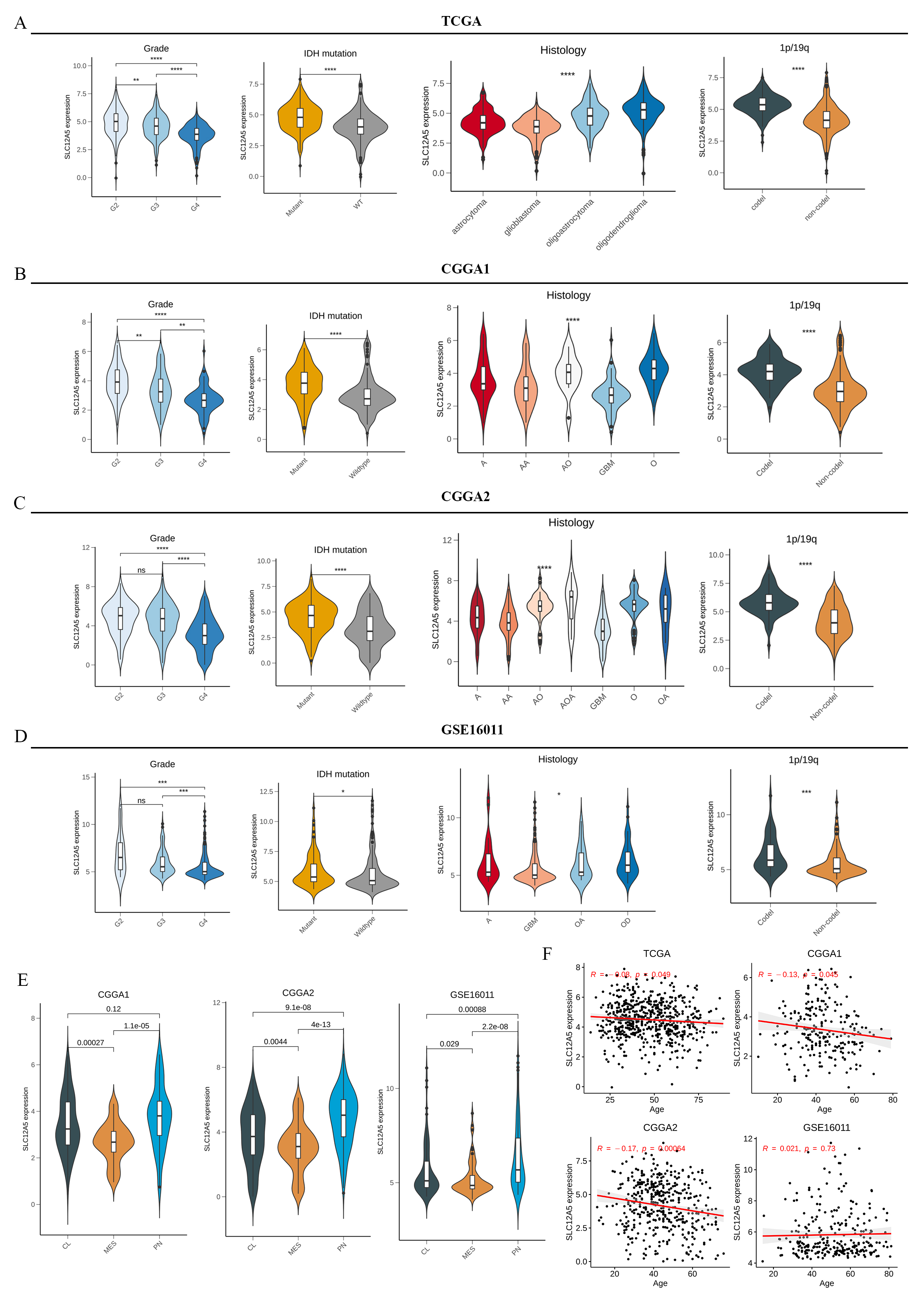

Supplement: Supplementary file 1 — Figure S1. [file JCMM-28-e18352-s001.jpg]

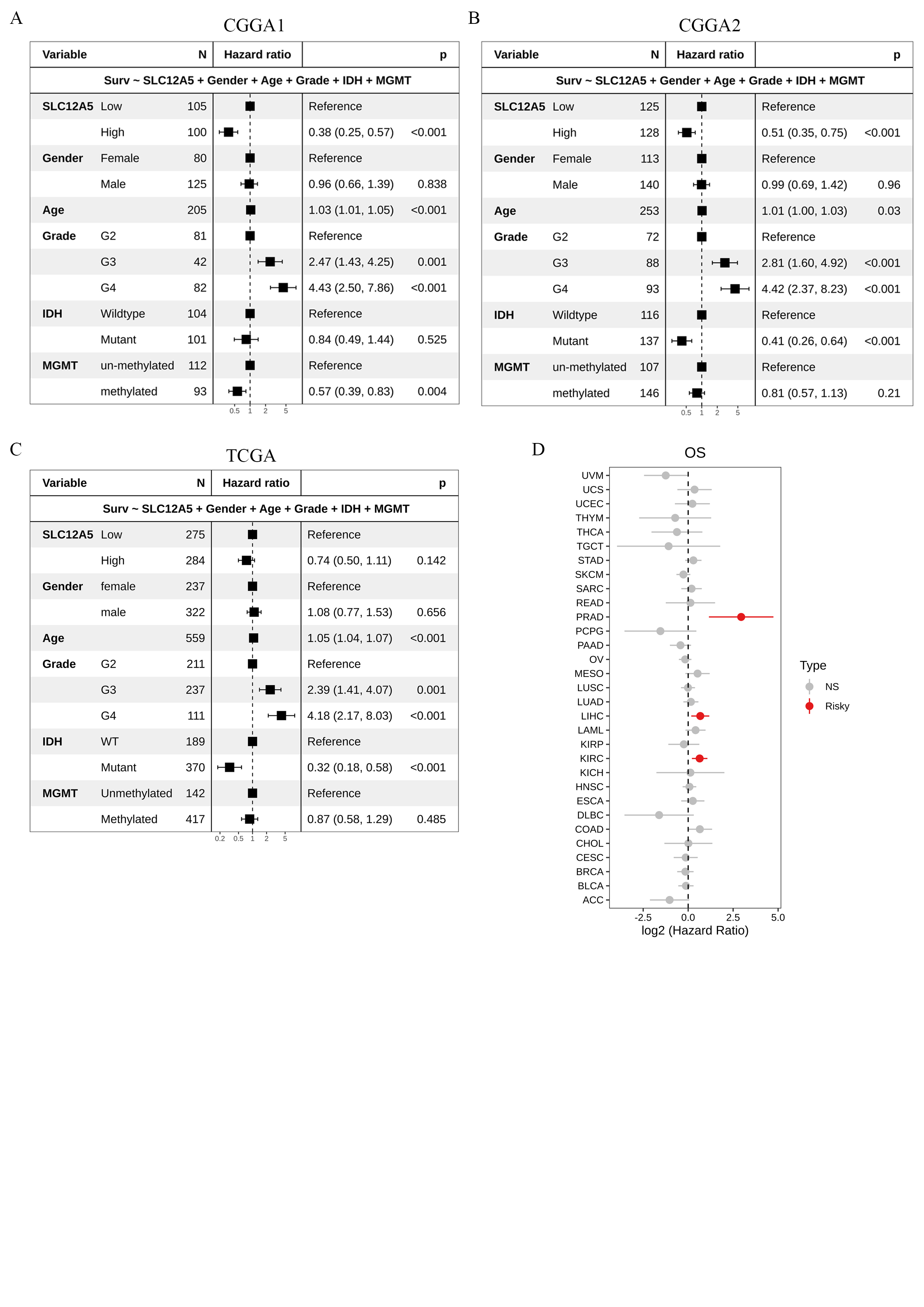

Supplement: Supplementary file 2 — Figure S2. [file JCMM-28-e18352-s002.jpg]

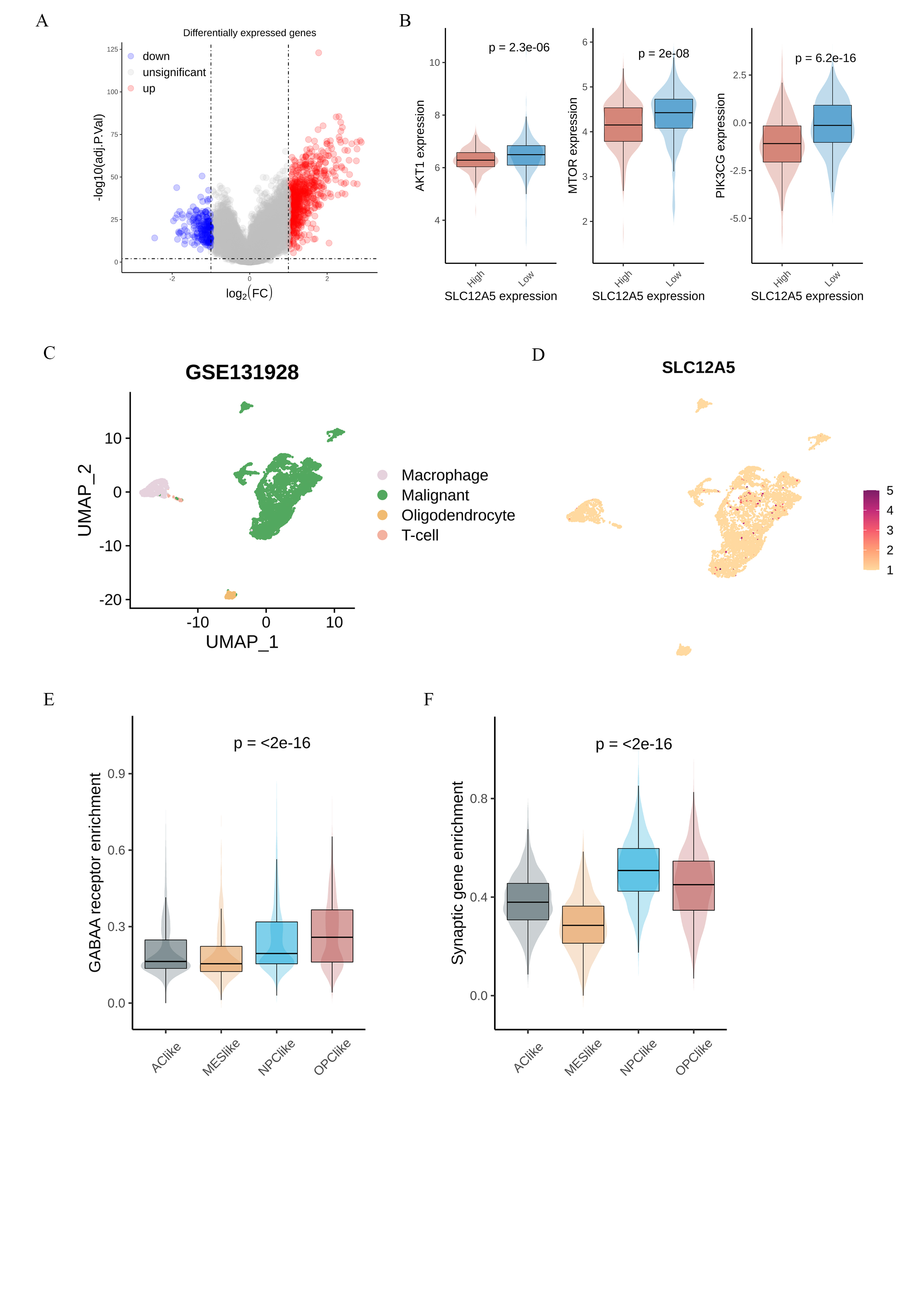

Supplement: Supplementary file 3 — Figure S3. [file JCMM-28-e18352-s004.jpg]

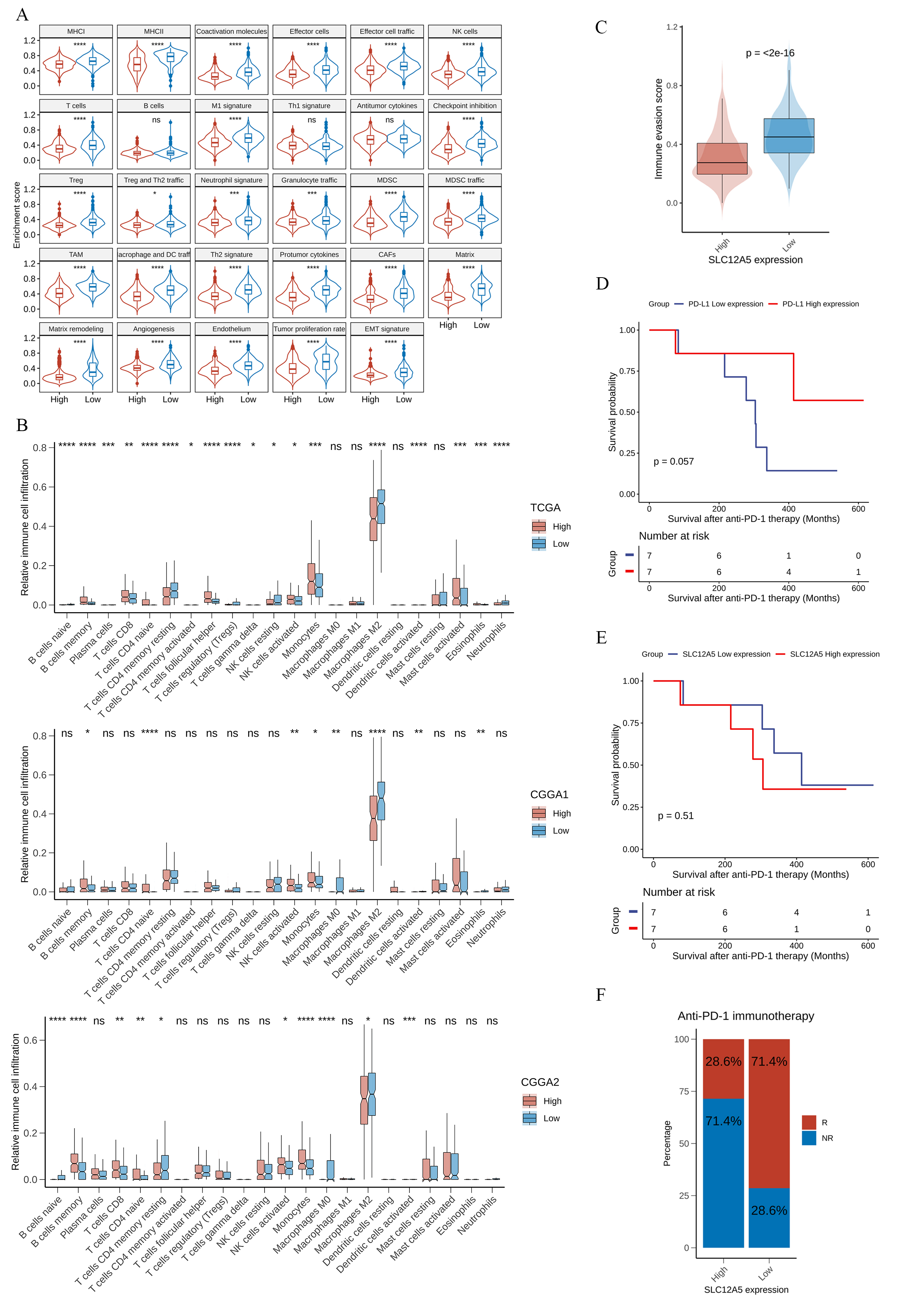

Supplement: Supplementary file 4 — Figure S4. [file JCMM-28-e18352-s003.jpg]
